# Perspectives and Requirements of Patients with Prostate Cancer on Mobile Health Interventions During Androgen Deprivation Therapy: A Descriptive Qualitative Study

**DOI:** 10.1111/jocn.17718

**Published:** 2025-03-09

**Authors:** Hongfan Yin, Chen Pan, Jia Gu, Yan Yang

**Affiliations:** ^1^ Department of Nursing, Renji Hospital School of Medicine, Shanghai Jiao Tong University Shanghai China; ^2^ School of Nursing Shanghai Jiao Tong University Shanghai China; ^3^ Department of Breast Surgery, Renji Hospital School of Medicine, Shanghai Jiao Tong University Shanghai China

**Keywords:** androgen deprivation therapy, mobile health, prostate cancer, qualitative study

## Abstract

**Aim:**

To explore the perceptions of patients with prostate cancer undergoing androgen deprivation therapy towards mobile health interventions.

**Design:**

The study employed a qualitative descriptive design.

**Methods:**

Seventeen participants were recruited from tertiary care hospitals from November 2022 to June 2023. The collected data were subsequently analysed using a content analysis approach.

**Results:**

The main themes were facilitators, barriers, information needs, emotional and social needs, and self‐management needs. Facilitators for using mobile health included support from healthcare professionals and family, competitive design features and user‐friendly interfaces. Barriers included negative past experiences with technology and a satisfactory current state of health. Informational support included those patients who expressed a desire for timely, accurate information integrated with traditional Chinese medical principles. Regarding emotional support, patients emphasised their independence, preferring not to burden family and friends, showing disinterest in mindfulness and relaxation therapies, and generally disliking online interactions with fellow patients. For self‐management, medication reminders and rehabilitation guidance were seen as vital tools to enhance supportive care.

**Conclusion:**

The study highlights the importance of customising mobile healthcare applications to meet unique needs among patients with prostate cancer and enhance their engagement and satisfaction. Understanding their specific preferences enables the development of more effective mobile healthcare applications.

**Reporting Method:**

The COREQ checklist.

**Summary**

- Implications for practice
  ○ This study offers insights for conducting mobile health research in developing countries like China and provides valuable guidance on elderly individuals' use of mobile health technologies.
- What does this paper contribute to the wider global clinical community?
  ○ This paper highlights the specific needs and preferences of these patients and underscores the importance of personalised healthcare solutions. Moreover, it encourages increased research into mHealth for elderly patients to improve their access to and use of these technologies.

## 1 Introduction

Prostate cancer, the second most common tumour globally after lung cancer, poses a significant health threat to men worldwide (Sung et al. 2021). Its incidence has risen sharply, particularly in China (Chen et al. 2016; Zheng et al. 2023). Androgen deprivation therapy (ADT) remains the primary treatment for advanced prostate cancer (Siegel et al. 2023), not only extending patient survival but also introducing complex physiological, psychological and social challenges (Donovan et al. 2015; Hanjing et al. 2023; Hormone Therapy for Prostate Cancer 2022). The rise of mobile health (mHealth) offers a promising solution, utilising mobile devices to improve access to health information, communication between patients and healthcare providers, and symptom monitoring (Nadal et al. 2020). Currently, there is limited research in China on the use of mHealth for patients with prostate cancer undergoing ADT, highlighting the necessity of understanding their perspectives and insights towards mHealth.

### 1.1 Background

Prostate cancer is a major global health issue with consistently high incidence rates. In 2023, the United States reported approximately 288,300 new cases of prostate cancer in men, comprising 29% of all male cancer diagnoses and marking it as the most diagnosed cancer in the nation (Siegel et al. 2023). In recent years, the incidence of prostate cancer in China has undergone significant changes, increasing tenfold over the past two decades, with an annual growth rate of 7.1%, making it the sixth most common cancer among men (Chen et al. 2016; Zheng et al. 2023). This rising trend is closely associated with an ageing population, increased PSA testing, and lifestyle changes linked to urbanisation and dietary transitions (Arigbede et al. 2024). Unlike in Western countries, where early detection is widespread (Sharma 2019), prostate cancer in China is often diagnosed at advanced stages, with approximately 70% of cases identified at later stages (Shanshan and Dingwei 2022). This difference is primarily due to lower awareness of screening, limited access to routine PSA testing, and disparities in healthcare infrastructure between urban and rural areas (Arigbede et al. 2024).

Androgen deprivation therapy, also known as endocrine therapy, constitutes the cornerstone of first‐line treatment for advanced prostate cancer (Siegel et al. 2023). ADT has been shown to significantly extend patient survival, with 5‐year survival rates reaching up to 97% (Siegel et al. 2023). This advancement implies that an increasing number of patients are living longer with the disease. However, despite improved survival outcomes, patients with prostate cancer in China face distinct challenges in long‐term disease management. Unlike Western settings where digital health interventions have been widely adopted, mHealth solutions tailored for patients with prostate cancer in China remain underdeveloped. Factors such as low digital literacy among elderly patients and uneven access to healthcare services contribute to the complexity of integrating mHealth into prostate cancer care (Li et al. 2023; Wang et al. 2024; Xu et al. 2024).

In addition to these issues, ADT presents complex challenges for patients. Physiologically, patients experience symptoms like hot flashes, sexual dysfunction, osteoporosis and hepatic anomalies. Psychologically, they grapple with depression, recurrence fears and uncertainty. Socially, they face familial strain, altered spousal dynamics and possible social discrimination (Donovan et al. 2015; Hanjing et al. 2023; Hormone Therapy for Prostate Cancer 2022). These considerations underscore the necessity for comprehensive management strategies that address the full spectrum of patients' experiences during and beyond treatment.

In the context of the burgeoning mobile Internet and widespread adoption of smartphones, mHealth has increasingly become integrated into various healthcare sectors (Nadal et al. 2020). mHealth, a subset of eHealth, refers to the use of mobile devices such as smartphones, tablets and wearable technologies to support healthcare delivery and health monitoring (Nadal et al. 2020). It offers an innovative and effective approach to patient care. mHealth enables symptom tracking, chronic disease management and medication reminders (Hamine et al. 2015; Islam et al. 2014; Link 2024). By facilitating health data exchange, it improves accessibility, supports patients' self‐management and enhances efficiency, making it particularly valuable for remote and chronic disease care (Howell et al. 2024; Samadbeik et al. 2023).

In cancer care, mHealth has demonstrated significant potential by addressing the complex physical, psychological and social needs of patients. These tools provide tailored education and symptom management, reduce anxiety and depression, and improve self‐management skills, ultimately enhancing the quality of life for patients with cancer (Samadbeik et al. 2023; Sun et al. 2024). In prostate cancer care, specifically, mHealth is particularly valuable for addressing treatment‐related challenges. For example, gamification‐based applications and educational tools have been linked to better treatment adherence and patient satisfaction, whereas symptom tracking apps provide holistic assessments that alleviate treatment‐related anxiety (Anjos et al. 2024; Homewood et al. 2024). Given the multifaceted challenges faced by patients with prostate cancer undergoing ADT—including physical symptoms like hot flashes, psychological distress such as recurrence fears, and social strains—there is a pressing need to align mHealth functionalities with these unique patient needs. Understanding their specific requirements, preferences and potential barriers is essential to design effective mHealth solutions tailored for this population.

Recognising the diverse needs of patients using mHealth solutions, it becomes essential to adopt a user‐centred approach in the development of these technologies. User‐centred design (UCD) is a pivotal approach in mHealth development, strongly endorsed by the World Health Organization (2011). This methodology emphasises engaging with end‐users and prioritising their needs throughout the development process, which typically involves four iterative phases: understanding the user context, identifying user requirements, designing solutions and evaluating outcomes (IxDF 2016). UCD has proven effective in oncology mHealth apps. For example, an app developed with UCD, involving patients with paediatric cancer and their parents, showed strong usability and helped improve antiemetic treatments by collecting nausea data (Eliasen et al. 2020). Similarly, the Lion‐App enabled patients with cancer to assess their quality of life with high usability and engagement through iterative user testing (Beutter et al. 2023). These examples highlight how UCD principles—patient feedback and iterative optimisation—lead to clinically effective, user‐friendly tools. Such insights are vital for designing mHealth solutions for patients with prostate cancer on ADT, where usability and functionality are key. By incorporating these principles at every stage, mHealth applications can significantly enhance the effectiveness of interventions and ensure alignment with users' needs and expectations (Farao et al. 2020; McCurdie et al. 2012).

Accordingly, this study utilised semi‐structured interviews with 17 patients with prostate cancer undergoing ADT, recruited through purposeful sampling in a large, top‐tier hospital in Shanghai, China, to gain an in‐depth understanding of their specific needs and input for mHealth‐based supportive care. Semi‐structured interviews were chosen because of their ability to balance structure with flexibility (Kallio et al. 2016), making them particularly effective for exploring complex and sensitive topics. Unlike structured questionnaires, which limit responses to predefined options, semi‐structured interviews allow interviewers to probe deeper into participants' perceptions and needs through follow‐up questions (Hardon et al. 2004; Polit and Beck 2010; Rubin and Rubin 2011). Furthermore, compared with focus groups, where social dynamics may inhibit personal expression, semi‐structured interviews provide a more individualised and adaptive approach, ensuring that participants can freely discuss their experiences without external influence (Cohen 2006). Given the exploratory nature of this study, this method is particularly well suited for capturing nuanced perspectives on mobile health interventions. The insights from these interviews directly inform the implementation of mHealth for ADT‐treated patients with prostate cancer. They reveal patient perceptions, identify key facilitators and barriers, and clarify needs related to information access, emotional support and self‐management. These findings provide the basis for strategies aimed at improving mHealth adoption and guiding the development of functionalities that align with patient needs, ensuring practical and relevant solutions.

### 1.2 Aim

The study aimed to explore the perceptions and needs of patients with prostate cancer undergoing androgen deprivation therapy towards mobile health interventions.

## 2 Methods

### 2.1 Design

A descriptive qualitative research approach was employed, utilising semi‐structured interviews to gather data. Semi‐structured interviews were chosen for their flexibility in exploring participants' perceptions of mobile health interventions. This study forms a segment of a broader study into mHealth interventions for patients with prostate cancer on ADT.

Guided by UCD principles, this study focused on integrating patient needs and perspectives at every stage. A flexible, semi‐structured interview approach was used without a predefined mHealth framework, allowing patients to freely express their views and envision ideal mHealth functionalities. Topics of interest, such as wearable devices, were explored by tailoring follow‐up questions to patients' preferences. The research prioritised patient experiences and needs, with data collection and analysis centred solely on patient interviews. This ensures that the findings authentically reflect patient needs, providing valuable insights for developing mHealth interventions for patients with prostate cancer.

The outline of the interviews was discussed and developed by our research team. To refine the interview outline, two ADT‐treated patients with prostate cancer with different demographic and technological backgrounds were selected for pre‐interviews. Patient 1 (59 years old, junior high education, diagnosed 2 years ago) had 15 years of smartphone experience and used it for about 6 h daily. Patient 2 (82 years old, university degree, diagnosed 10 years ago) had 3 years of smartphone experience and used it for less than an hour daily. Their feedback helped enhance the clarity, relevance and comprehensiveness of the outline, which was then finalised for formal use. The revised interview outline is as follows: (1) Can you describe any difficulties or particular needs you have faced during ADT for prostate cancer? How have these impacted your treatment and daily life? (2) When utilising your smartphone, which specific functions do you frequently use, and what motivates you to use them? Additionally, have you encountered any challenges while using these functions? (3) Reflecting on your usage of a smartphone, what specific functions do you utilise in relation to managing your disease? Can you elaborate on your reasons for using these functions and any challenges you've encountered? Do you ever feel frustrated, anxious or stressed while managing your health through smartphone apps? Can you share specific examples? (4) Had you been aware of mHealth applications for prostate cancer prior to this interview? Can you share your perceptions or any information you might have come across about these apps? (5) If there were an mHealth application developed for prostate cancer management, would you consider using it? Please explain why you would or would not be interested in such an application. (6) What features and resources would you like to have in an mHealth application tailored for prostate cancer? What needs do you think should be addressed by the app, and do you have any specific suggestions? Would you find it helpful if the app included resources or features to address emotional or psychological challenges during your treatment? If so, what kind of support would you like? (7) Do you have any other thoughts or insights regarding the use of mHealth in the context of prostate cancer that you would like to share?

### 2.2 Study Setting and Sampling

Purposeful sampling was conducted in a large, top‐tier hospital in Shanghai, China. This approach involved selecting patients with diverse background characteristics, including different age groups, educational levels, types of medication, duration of illness and severity of the condition for interviews. A urologist and a nurse assessed the eligibility of the patients for the study by screening their electronic medical records. Inclusion criteria were as follows: (1) pathologically diagnosed with prostate cancer and receiving ADT; (2) clear consciousness, no barriers to verbal communication and able to express views accurately; (3) informed consent and willingness to participate voluntarily; and (4) ownership of a personal smart mobile electronic device. Exclusion criteria were as follows: (1) known other malignant tumours that are progressing or requiring current treatment and (2) known symptomatic central nervous system metastases and/or carcinomatous meningitis. Patients with other malignant tumours were excluded to avoid confounding factors unrelated to androgen deprivation therapy, as these patients may face additional health priorities or symptoms that could obscure the focus on prostate cancer‐specific mHealth needs. Similarly, patients with symptomatic central nervous system (CNS) metastases or carcinomatous meningitis were excluded because of the potential impact of these conditions on cognitive function and communication abilities, which are critical for qualitative data collection and analysis.

In this study, an invitation was extended to 148 patients, selected based on predefined inclusion and exclusion criteria, to participate in a qualitative research project. The researcher initiated contact through telephone communications to elucidate the study's objectives, methodologies and overarching significance, ensuring a clear understanding before obtaining informed consent. Appointments for the interviews were meticulously scheduled, considering the convenience of the interviewees; settings within the hospital ward were strategically chosen for their tranquillity and privacy; these included unoccupied meeting rooms and consultation rooms. The recruitment process was carefully managed and monitored until a heterogeneous sample was curated, showcasing a wide range of variability in age, treatment stages, levels of digital health literacy and general education levels, thereby reaching a point of data saturation.

Out of 148 patients who were invited, a total of 29 consented to participate in the study. However, among these 29 consenting participants, there were instances of non‐participation: three patients did not attend as per the scheduled times, two were excluded because of unforeseen personal emergencies, and seven could not be reached by telephone following the initial acceptance. Data collection and analysis were conducted simultaneously, allowing for an iterative approach to identify emerging themes. After 15 interviews, data saturation was reached, as no new themes emerged. To confirm the robustness of the findings, two additional interviews were conducted, validating the consistency and comprehensiveness of the identified themes. The final sample consisted of 17 participants.

### 2.3 Data Collection

Between November 2022 and June 2023, the first author (H.Y., female) and the co‐author (J.G., male) conducted face‐to‐face interviews with the patients. They are both currently master's students and have completed a 6‐month offline course and a 6‐month online course in qualitative interviewing. Prior to the interviews, patients provided informed consent and completed two brief questionnaires, designed to achieve maximum variation sampling and to contextualise the qualitative findings. The first questionnaire gathered general demographic information, including age, education level, marital status, number of children, occupation and monthly income. These variables were essential to ensure diversity among participants and to capture a wide range of perspectives. For instance, differences in age, marital status and income were selected to explore how different life stages or financial circumstances might shape patients' access to and use of mHealth solutions. The second questionnaire focused on disease‐specific details, encompassing the diagnosis date of prostate cancer, duration of ADT and medications used for endocrine treatment. This allowed us to include patients at different stages of their disease trajectory (e.g., newly diagnosed versus long‐term survivors) and those on various endocrine therapies, providing a broader understanding of how treatment contexts influenced their mHealth‐related perceptions. The data were collected using a recording device during the interviews. Using open‐ended questions as an introduction, adjusting the sequence of questions flexibly according to specific circumstances, and employing techniques such as repetition, probing and clarification, ensured that interviewees fully articulate their thoughts. This approach aimed to maximise the comprehensiveness and authenticity of the data. During interviews, attentive listening is emphasised, along with the use of verbal and non‐verbal techniques to alleviate patient nervousness. Additionally, close observation and timely documentation of interviewees' non‐verbal behaviours (gestures, expressions, etc.) are conducted to avoid inducing behaviours. After the interview, the contents of the interview recordings will be provided to the participants for feedback, inviting them to revise or supplement as needed. The duration of the interviews with patients ranged from 17 to 93 min, averaging 29 min. The interviews were meticulously transcribed verbatim, resulting in transcripts totalling 115,932 Chinese characters.

### 2.4 Data Analysis

The study employed qualitative content analysis (Graneheim and Lundman 2004), operationalised through NVIVO 12.0 software, to systematically analyse both manifest and latent content. The process began with repeated readings of the transcribed data to gain a comprehensive understanding of the material. The data were then segmented into meaning units—words, phrases or sentences—and coded descriptively to capture the explicit content. A comparative analysis of these codes identified similarities and differences, forming categories that represented observable patterns, such as recurring behaviours, statements or needs. For latent content, the team analysed these categories for deeper meanings, uncovering underlying motivations, socio‐cultural influences and assumptions. These were synthesised into themes that provided broader insights. Regular team discussions ensured consensus on the interpretation of both manifest and latent content, minimising bias and enhancing the credibility of the findings.

Two researchers, H.Y. and J.G., independently performed the coding. Consensus on coding and entries was achieved through repeated team discussions. Where discrepancies arose, C.P. (female, MD, nurse) and Y.Y. (female, PhD, supervisor) provided adjudication. This process yielded five themes. The translation process used a bidirectional approach for semantic accuracy and cultural relevance. Two bilingual researchers, with expertise in qualitative research (one in medicine, the other in nursing), translated the quotations from Chinese to English. These translations were then back‐translated by two independent bilingual individuals, one with 7 years of experience in an English‐speaking country and the other with 12 years of experience. This allowed for comparison with the original text to ensure consistency. Finally, a research team reviewed the translations to preserve participants' intended meanings and ensure clarity for English readers. This process maintained the authenticity and cultural appropriateness of the translations.

### 2.5 Ethical Considerations

The study was approved by the ethical review of the School of Public Health and the School of Nursing of Shanghai Jiao Tong University (SJUPN‐HY‐202312‐23‐KS1).

### 2.6 Rigour and Reflexivity

To enhance the rigour of the study and minimise biases, several measures were implemented. Three researchers underwent training in qualitative research methods prior to the study to ensure effective data collection and analysis. Researcher bias was minimised through collaborative analysis, with two researchers independently coding the data, followed by team discussions to refine the results. Sampling bias was addressed through purposive sampling, with participants selected to represent a variety of backgrounds, including differences in age, education, treatment duration and mobile technology usage. To mitigate social desirability bias, participant anonymity was ensured, and a neutral, supportive interview environment was created to encourage honest responses and reduce the likelihood of answer adjustment.

## 3 Results

### 3.1 Characteristics of Study Subjects

Seventeen patients undergoing ADT for prostate cancer were ultimately included in the study (Table 1 and Table S1). The mean age of participants was 71.8 years, with a range of 61 to 84 years. The average reported duration since diagnosis was 3 years. Participants had been utilising the internet for an average of 8.3 years and reported an average daily internet usage of 3 h. A majority of 52.9% (9 out of 17) indicated their willingness to adopt mHealth solutions for managing their condition.

**TABLE 1:** General demographic data of patients with prostate cancer undergoing ADT.

Patient	Age	Education level	Duration of illness	Duration of smartphone usage (years)	Daily internet usage (h)	Willingness to use mobile health
N1	61	Community college	< 1 year	10	4	Willing
N2	71	Middle school	2 years	5	3	Willing
N3	73	Vocational high school	8 years	9	2	Willing
N4	70	Undergraduate	< 1 year	5	2	Willing
N5	66	Community college	< 1 year	10	4	Unwilling
N6	66	Community college	3 years	8	7	Unwilling
N7	76	Middle school	9 years	7	2	Willing
N8	65	Middle school	2 years	13	5	Willing
N9	70	Middle school	9 years	13	6	Unwilling
N10	71	Elementary school	7 years	3	0.5	Willing
N11	80	High school	2 years	13	3	Unwilling
N12	74	Undergraduate	4 years	13	4	Willing
N13	74	Undergraduate	2 years	13	2	Willing
N14	70	Middle school	4 years	4	1	Unwilling
N15	81	Middle school	4 years	5	3	Unwilling
N16	84	Middle school	1 year	7	2	Willing
N17	68	Middle school	3 years	6	2	Unwilling

### 3.2 Patients' Experience With Mobile Devices

Among 17 patients, the majority (15 out of 17) utilised various smart functionalities of smartphones beyond basic calling and texting, akin to the use of feature phones. Some even employed wearable devices to monitor physiological parameters. These individuals integrate such technologies into their daily routines for accessing information, entertainment, social interaction and health management. They view these devices as a means of emotional relief during times of solitude, indicative of the integral role mobile technology plays in their lives.

Within the cohort, 15 individuals used WeChat—a widely used communication app in China similar to WhatsApp—for text messaging, voice and video calls. This app also features a fitness component, which 12 patients used to monitor their daily steps and exercise activities, sharing and comparing with friends. Nine patients frequented mobile news apps, eight browsed short videos on TikTok, seven researched health‐related information via platforms like Baidu (a major Chinese search engine similar to Google) and Weibo (a Chinese micro‐blogging platform similar to Twitter), and six made purchases of snacks and clothing using e‐commerce apps. Three owned wearable devices.

Most patients were able to use straightforward apps like WeChat, TikTok and news applications with ease. Nearly half were proficient in more complex digital interactions such as online shopping and social media engagement on platforms like Weibo. Particularly noteworthy was one patient who adeptly used a high‐end wearable device with mobile applications to continuously monitor vital health statistics, including breathing, heart rate, mood, blood pressure and glucose levels.

The main themes were facilitators, barriers, information needs, emotional and social needs, and self‐management needs.

### 3.3 Facilitators in Mobile Health for Prostate Cancer Undergoing ADT

Table 2 describes the factors that enhance mHealth utilisation among patients undergoing ADT for prostate cancer.

**TABLE 2:** Facilitators for the use of mHealth.

Sub‐themes	Description
Formal development and trusted support	Mobile health apps, developed by trusted medical staff through formal processes, ensure privacy and offer reliable, timely support recommended by healthcare professionals and family
Ease of use	The apps are favoured for objective measurement and have a simple login, click and operation process
Competition	Patients enjoy products with competitive features, such as comparing step counts with friends, which promotes the use of mobile technology

Endorsement and instructive backing from kin and healthcare experts emerge as a cornerstone, fostering confidence in mHealth solutions. Participant N1 highlighted the significance of support, ‘I definitely hope to receive guidance from medical personnel and my daughter when using mobile health applications, especially when I'm unsure about certain functions. When it comes to downloading apps, I rely entirely on my daughter's judgment. If she believes an app is useful, I will download it; if she advises against it, I won't proceed’.

The development of mHealth apps by trusted medical experts is key to increasing user trust and protecting privacy. Participant N3 stresses the importance of trustworthy development, saying, ‘Apps developed in a hospital setting give me confidence. I want nothing akin to an informal, ‘street stall’ product’.

The ease of use emerged as a key facilitator in the adoption of mobile health technologies among our participants. They expressed a desire for applications with simple nested structures and user‐friendly operations. This preference is particularly important for elderly patients, who may not be as adept at navigating complex digital interfaces.

Participant N3 articulated this need for simplicity, ‘As an older person, I'm not as skilled with smartphones like the younger folks. I can manage opening an app and simple clicks, but if it gets too complicated, like having to click multiple times and then make selections, I struggle to do it on my own’.

Among the nine patients who were receptive to using mobile health technologies, eight expressed a preference for objective measurement methods that require minimal effort on their part. Wearable devices that are capable of passively collecting data were particularly favoured, as they eliminate the need for manual data entry and allow for continuous monitoring. Participant N12 exemplified this sentiment by stating, ‘I like passive monitoring. Wearing an electronic watch that requires no active input from me and automatically tracks my condition 24 h a day is very convenient for me’. This inclination towards devices that facilitate passive data collection suggests that the perceived ease of use and the ability to gather health data unobtrusively are significant factors in the willingness of patients to engage with mobile health technologies.

Many patients enjoyed products that incorporated competitive elements, such as the ability to compare step counts with friends. Specifically, 12 out of 15 patients (80%) who used WeChat utilised its fitness component for this purpose. This gamified approach appears to motivate patients to use the technology more consistently by tapping into their social networks and encouraging a healthy competition. For instance, Participant N4 articulated their appreciation for this aspect, stating, ‘I enjoy monitoring the step count on WeChat. It motivates me to move a bit more each day to keep up with—or surpass—my friends' activity levels’. Participant 1 also emphasised the importance of social comparison in mHealth design, stating, ‘I hope that your product allows me to see how my data compares with other patients. For example, knowing my ranking among them would help keep me motivated’. This finding underscores the value of integrating gamified features into mHealth applications, as such designs can enhance sustained engagement and encourage healthier behaviours.

### 3.4 Barriers in Mobile Health for Prostate Cancer Undergoing ADT

Table 3 outlines the barriers to mHealth utilisation among patients undergoing ADT for prostate cancer.

**TABLE 3:** Barriers for the use of mHealth.

Sub‐themes	Description
No demand	There is no perceived need due to long‐term adaptation to prostate cancer or satisfaction with current treatment methods
Smart device challenges	Elderly patients have less experience with apps and often believe they can't use them
Past negative experiences	Past negative experiences with mobile technology can be deterrents

A significant barrier identified in the adoption of mobile health technologies among the participants was the lack of perceived necessity. This perception primarily stems from the prolonged duration of living with prostate cancer, during which patients have incrementally adapted to the disease‐related challenges. Through this adaptation process, many have developed individual strategies to manage adverse reactions, reducing their reliance on external health resources. Participant N9 articulated this perspective: ‘At first, when I was just diagnosed, I had so many questions and nowhere to get answers. But now, after living with this for years, I don't really see the need for extra stuff like mobile health. I have my regular check‐ups every three months, and that works for me’.

Another factor influencing the reduced demand for mobile health solutions among patients is the mild nature of adverse reactions to ADT. This has led to a general satisfaction with the treatment and a perceived lack of issues requiring resolution. As expressed by Participant N11, ‘I haven't experienced any notable side effects; the treatment hasn't really had any negative impact on me, so there aren't any specific issues I feel the need to address’.

In addition, challenges associated with smart device usage emerged as a significant barrier, especially among older patients. This group often uses apps sparingly and tends to believe they lack the necessary abilities to effectively utilise such applications. Participant N6 illustrated this barrier: ‘I'm hesitant to use mobile health apps for prostate cancer since I seldom use apps in general. Plus, at my age, I don't really feel up to learning something new like this’.

Furthermore, previous negative experiences with mHealth can contribute to a reluctance to use apps. Issues such as inaccurate measurements and complicated setups have led some patients to view apps as potentially troublesome. For instance, Participant N14 shared their frustration: ‘I don't want to use these apps. We have a smart device at home, and it was such a hassle to set up (frowns and shows signs of irritation). It's unreliable too, sometimes it starts making noise when you don't want it to, which is really annoying’. Similarly, Participant N17 recounted a specific issue with a smartwatch: ‘I used to have a smartwatch, but I stopped wearing it because the measurements were inaccurate’.

### 3.5 Information Needs for mHealth Among Patients Undergoing ADT for Prostate Cancer

Table 4 presents a detailed overview of the information needs for mHealth among patients undergoing ADT for prostate cancer.

**TABLE 4:** Patients' information needs for mHealth.

Sub‐themes	Description
Early disease provision	Patients have the most urgent need for information when they are first diagnosed with a disease
Difficulty in obtaining reliable information	No channels for obtaining reliable information, lack of ability to discern the truth of information and doctors not having time to answer
Positive, gentle expression	Fear of overly direct and serious expression methods, hoping to be soothed and have emotional needs valued when receiving information
Chinese traditional medicine	Patients express a strong interest in information related to traditional Chinese medicine and traditional health preservation

In the cohort of patients undergoing ADT for prostate cancer, there was a particularly urgent need for information during the early stages of their condition. We found that patients with a longer duration since diagnosis generally reported that their need for information was not as intense or pressing as it was at the time of their initial diagnosis. Participant N7 reflected, ‘When I was first diagnosed, I was extremely anxious and frantically searched everywhere for information. Now, as I have gradually become more accustomed to managing the disease, I feel somewhat better’. However, due to the evolving nature of ADT, which often requires regular adjustments in treatment plans, new uncertainties continue to emerge. Participant N12 shared, ‘I've been diagnosed for four years now, and there's still so much I have to learn. Each new phase of treatment introduces its own set of challenges and questions, such as adapting to new medications and understanding their effects on my body’.

Patients demonstrated varied preferences in seeking information, reflecting the diversity of their habits and needs. 52.9% (9/17) of patients preferred using mobile news apps, whereas 47.1% (8/17) browsed short videos on TikTok and 41.2% (7/17) used platforms like Baidu and Weibo to search for health‐related information. However, many patients expressed difficulty in discerning the credibility of online health information. Participant N4 shared his frustration, ‘I try to find information about prostate cancer on Baidu, but it's hard to tell what's true and what's not, and we elderly are often deceived’. Additionally, the busy schedules of healthcare professionals limit the opportunity for patients to obtain comprehensive information. Participant N2 expressed, ‘The doctors just tell me to undergo tests, take injections, or medications without explaining why. They see so many patients that I only get a few minutes. I have many questions but hesitate to disturb them. Sometimes, if I ask more, I feel brushed off’. These findings highlight both the challenge of finding trustworthy health information and the diverse channels patients use to seek it. To meet these varied preferences, mHealth applications could provide information in multiple formats—for example, text‐based content for those who prefer reading, short videos for visual learners. By aligning with patients' individual habits and preferences, mHealth solutions can enhance both the accessibility and trustworthiness of health information.

Moreover, some online information is overly aggressive and frightening, potentially causing intense fear and psychological trauma. Many participants highlighted concerns about distressing online content and its negative psychological impact. Participant 10 shared a concern about the tone of health information, emphasising that if the healthcare team could provide educational content through the mHealth tool, it would be very helpful. However, they emphasised that the tone should be softer and more reassuring to avoid causing fear. As Participant 10 stated, ‘It would be really convenient if you provided health information in the mHealth, but please make it a little softer, because some descriptions are too hopeless and frightening’. Similarly, Participant N12 described how shocking and terrifying online information can be, stating, ‘Sometimes the information online is shocking, like the dire outcomes of late‐stage prostate cancer. Such content is too terrifying; after reading it, I can't sleep at night’. These insights suggest that mHealth solutions need to focus not only on providing accurate information but also on delivering it in a compassionate, supportive manner to reduce anxiety and foster a positive patient experience.

In this context, patients undergoing ADT for prostate cancer are increasingly turning to mobile medical solutions, seeking timely and professional support through online channels. In addition to disease and treatment‐related information, we observed that 82% (14/17) patients have a profound interest in Traditional Chinese Medicine (TCM) and wellness practices. Participant N13 discussed his confusion about integrating TCM into his treatment, ‘The Chinese doctor diagnosed me with deficiencies in qi and blood, as well as kidney deficiency. I was curious to see if TCM could help balance my body, but my Western doctor advised against it, leaving me puzzled’. Another participant, N1, inquired about dietary habits conducive to health recovery, ‘I consume a sea cucumber daily to nourish my body. What else can I eat to enhance my recovery?’ N17 expressed a desire for clear and reliable information on these matters, saying: ‘If we have any confusion, such as about TCM or what supplements to take, it would be great to have a platform that provides us with accurate information’.

### 3.6 Emotional and Social Needs

Table 5 describes the emotional and social needs associated with mobile health for patients.

**TABLE 5:** Patients' emotional and social needs for mHealth.

Sub‐themes	Description
Heavy emotional burden	Patients exhibited notable feelings of shame and uncertainty about their disease
Stoicism and strong independence	Elderly male patients demonstrated stoicism and a strong sense of independence, accustomed to facing problems alone and reluctant to seek help from friends and family
Reluctance to seek help from friends and family	These patients showed a reluctance to seek help or share their concerns with friends and family
Disinterest in emotional support	Patients showed little interest in emotional support therapies like mindfulness and relaxation methods, generally rejecting them
Inconsistent attitudes towards peer communication in mobile health	Most patients were not interested in engaging in peer communication, whereas a few expressed a desire for the establishment of such functionality

During ADT, patients with prostate cancer experience a range of negative emotions and social issues. Participant N9 shared, ‘I keep my illness a secret as much as possible. Those around me who have this illness also hide it. After telling others, they avoid me, thinking it's contagious’. The diagnosis of cancer instils fear and apprehension about discrimination from peers, and the feminising symptoms (such as breast development) associated with ADT exacerbate their feelings of shame about the illness. Participant N10 discussed the challenges with physical changes, ‘The breast enlargement is really unseemly. Some say I look perverted. I always wear tight clothes when going out now because it looks so bad; it's a huge burden on my image’. Throughout the course of ADT, regular blood marker tests (typically every 3 months) are necessary to monitor disease control, which can be an excruciating process for them. Due to excessive worry about their indicators, they may even undergo multiple tests in different hospitals within a few days to verify their levels. Participant N2 expressed his anxiety, ‘I get very nervous and scared for the PSA test every 3 months. Any slight change, and I feel a huge psychological burden. I'm terrified of what the disease might turn into later (cries)’. Even minor increases within normal ranges can cause them considerable distress.

Despite their significant emotional needs, 86.4% (13/17) of the patients refuse help from friends and family, and are even reluctant to discuss related topics with them. Participant N11 explained his perspective, ‘I've never talked about my pain; all my relatives and friends are unaware of it. I don't think it's necessary to tell them because they can't really help me’. This refusal to accept help may stem from their traits of stoicism and independence. Many patients believe that as the pillars or elders of their families, they should maintain a strong facade, even in the face of life's most challenging trials. They feel that showing any vulnerability in front of their loved ones is unacceptable, leading them to conceal their fears and suffering. Participant N7 shared his experience, ‘It's difficult to talk about it with my family. Doing so would only add to their worries. They are already busy with their work and I don't want to burden them. It's not easy to discuss. My spouse would be extremely worried and scared’. Patients also place a high value on their independence, often taking on responsibility and managing their treatment and health by themselves. Participant N10's comment highlights this attitude, ‘No need for that. I can handle it on my own. Don't worry about me’. Participant N13 described how he faced his illness alone, ‘From the time I found out about my illness to the diagnosis and preparing for the treatment plan, my wife knew nothing. I came here secretly, acting as if I was not sick. It was not until the hospital required a family member's presence for the surgery that I told her. I didn't want her to be as anxious as I was, hanging in suspense’.

Additionally, the patients show little to no interest in relaxation therapies such as meditation and mindfulness. This attitude likely stems from scepticism about the practical effectiveness of these methods, or because they do not align with their personal lifestyle preferences and habits. Participant N5 expressed his opinion, ‘Things like listening to music or meditation, I don't think they're necessary. I don't like them’. Participant N8 shared a similar view, ‘No need, they are useless’. This reflects a possibility that the patients may have insufficient knowledge of these newer relaxation techniques, or they feel that these methods are not suitable for their personal needs and circumstances.

On the contrary, many patients expressed a strong desire to communicate with healthcare professionals, but due to the busy schedules of these professionals, such in‐depth conversations are often difficult to achieve. Participant N2 joyfully said, ‘Today I am really, really happy, it's the happiest I've been all year. It's been such a long time since I've talked to anyone about my illness, I am so grateful to you all’. This reflects that despite their tendency towards stoicism and independence, they still yearn for meaningful exchanges with healthcare providers who understand their condition.

With the difficulty in accessing prolonged face‐to‐face consultations, many patients have turned to mobile health as a means to communicate and share their experiences with healthcare professionals, seeking support in their daily lives. At the same time, their attitudes towards engaging in peer communication through mobile health are complex and varied. The majority of patients feel that it is unnecessary to interact with strangers, and even find that more communication with other patients can be distressing, particularly when seeing others with worsening conditions, which amplifies their own fears and concerns about the future. Participant N6 expressed his feelings: ‘Well, I've had conversations with other patients before, and it was quite upsetting. Some of them aren't doing well in treatment, and their indicators keep rising. Hearing about it, I'm scared I might end up the same. And honestly, since we're all strangers, I don't see much need for that kind of interaction’. However, a small group of patients are very much in favour of organised peer support groups. They believe that mutual exchanges can offer opportunities for learning and helping each other, such as sharing ways to deal with symptoms like incontinence or communicating their treatment experiences. Participant N4 said: ‘I really like it. I've found my own ways to handle the side effects and often share these experiences with colleagues who are also sick. They find it helpful, and that makes me happy too’.

### 3.7 Self‐Management Needs

During the process of home care, a common issue frequently mentioned by patients is ‘frequent forgetfulness’. This forgetfulness encompasses various aspects, including forgetting to take medications and failing to complete prescribed rehabilitation exercises. Participant N7 explained, ‘For instance, with taking medications, sometimes I forget to take my Chinese medicine, and other times I forget my abiraterone (a prostate cancer hormone therapy drug). As I get older and the number of medications increases, it's really easy to forget, so I need reminders’. Additionally, patients also expressed difficulty in maintaining consistency with rehabilitation exercises recommended by their doctors, often forgetting to perform them. Participant N1 shared his experience, ‘I know I need to do daily pelvic floor exercises, but it's incredibly easy to forget, and no one is there to remind me’.

Therefore, they have a particular interest in using mobile health applications as a reminder to take their medications and perform rehabilitation exercises regularly. Participant N8 expressed, ‘I used to remind myself about my medications with handwritten notes. If I could get reminders through a mobile health app, that would be just perfect!’

## 4 Discussion

In prevailing discourse, it is often posited that the elderly face substantial challenges in utilising mobile technologies due to the deterioration of physical faculties (e.g., vision, attention, fine motor skills) and a constrained proficiency with internet and technological products (Busch et al. 2021; Duangpatra et al. 2021; Yang et al. 2022). However, this study presents a divergent view, particularly among a subset of the elderly male demographic, specifically patients with prostate cancer. The findings indicate a notably higher competency in this group, with approximately 88.2% demonstrating proficiency in using at least one smart application and exhibiting a positive disposition towards smartphones. This variance in technological adaptability may be partially attributed to the demographics’ geographic location in Shanghai, a relatively advanced region in China, coupled with a potential increase in smartphone dependency catalysed by the COVID‐19 pandemic (Chemnad et al. 2022; Wicaksono et al. 2023). Although elderly individuals have a positive attitude towards mobile technology, only 52.9% are willing to use mHealth for prostate cancer management. This highlights the need to explore factors influencing mHealth adoption for prostate cancer ADT.

### 4.1 Facilitators and Barriers

In exploring the factors that facilitate or hinder the adoption of mobile health technology by the elderly, this study underscores the significant roles of healthcare professionals and family members in promoting adoption. This finding aligns with the constructs of ‘Facilitating Conditions’ and ‘Social Influence’ in the Unified Theory of Acceptance and Use of Technology (UTAUT) (Venkatesh et al. 2003). Facilitating Conditions are especially important for older adults, as they often rely heavily on timely responses to their questions and step‐by‐step operational guidance. Similarly, Social Influence, particularly the attitudes and support from family members, frequently determines whether older adults accept and utilise mobile health technologies. Family approval and assistance often act as pivotal enablers, particularly in situations where elderly individuals face barriers to independent adoption.

These findings also resonate with the Senior Technology Acceptance Model (STAM), emphasising the critical role of environmental support and positive familial and social influences in shaping elderly users' technology acceptance behaviours (Chen and Chan 2014). Although the STAM highlights the importance of gerontechnology self‐efficacy—a term referring to older adults' confidence in using digital tools—our findings reveal that this confidence is often undermined by perceived complexity and prior negative experiences with mobile technologies. Such challenges lower motivation and diminish their engagement with new tools, underscoring the necessity of targeted interventions to enhance self‐efficacy.

Notably, the STAM posits that perceived ease of use is not a key determinant for elderly technology adoption. However, our findings diverge from this view, aligning more closely with the Technology Acceptance Model (TAM) (Davis 1985). Specifically, we found that perceived ease of use is a significant motivator for older adults adopting mobile health applications. Given the physical and cognitive limitations that often accompany ageing, intuitive and user‐friendly designs are essential to overcoming these barriers. Consequently, our development approach prioritises clear, accessible and straightforward interface designs that address the unique needs of older users.

Building upon these findings, several design elements have been prioritised to align with the unique needs of elderly users. It is recommended that the app be intuitive, user‐friendly and designed with ageing‐friendly principles in mind to enhance perceived ease of use. The interface should be simplified, and the app structured to make navigation as straightforward as possible, addressing the physical and cognitive limitations that often accompany ageing. Additionally, integrating healthcare professionals, such as trusted doctors and nurses, into the development process would help enhance the tool's credibility and ensure it meets patient expectations. These professionals can play a vital role in bridging the gap between technology and healthcare, providing necessary guidance and reassurance to foster adoption. Furthermore, involving family members during training and onboarding is suggested as essential to reducing adoption barriers, offering emotional support and providing practical assistance.

### 4.2 Information Needs

Information needs play a central role in fulfilling emotional and self‐management demands. Many patients express hesitancy towards emotional support interventions, such as psychological support therapies, partly due to their lack of understanding of these therapies' efficacy. Additionally, exposure to unverified counselling services or scams has led to distrust in emotional support, further hindering its acceptance. Bridging these information gaps by providing accurate and reliable health information is crucial for alleviating patients' emotional distress and creating conditions conducive to accepting emotional support. At the same time, the influence of information needs on self‐management demands cannot be overlooked. A lack of information about the importance and effectiveness of treatment plans and rehabilitation often leads to barriers in self‐management. Many patients fail to recognise the significance of adhering to medication schedules and maintaining rehabilitation exercises, which restricts their ability to sustain self‐management behaviours. Therefore, ensuring patients have timely access to key information is fundamental to fostering self‐management practices.

In the process of providing informational support, we observed a remarkable phenomenon where nearly all patients inquired about the significant role and implications of TCM. TCM has deep cultural roots in China, using treatments such as acupuncture and cupping, and holds an important position in both Chinese culture and the contemporary healthcare system (The Editors of Encyclopædia Britannica 2023). However, public opinion on TCM is highly polarised (Xin et al. 2020). Due to the fundamental differences in philosophy and methods between TCM and Western medicine, patients often experience conflict and confusion. Strong denial by Western medicine experts can lead to further conflict and doubts among patients. To address this issue, mHealth platforms can act as a bridge by integrating scientifically validated TCM information with Western medical guidelines. Moving forward, we aim to incorporate high‐quality, evidence‐based content from both TCM and Western medicine, ensuring that educational materials undergo peer review by experts in both fields. Additionally, we propose establishing backend support from both TCM and Western medicine professionals, allowing patients to consult with experts in real‐time for more personalised guidance. This approach will help enhance the credibility, accuracy and comprehensiveness of health information, offering a balanced view that can cater to patients' diverse needs and cultural backgrounds.

It is crucial to deliver information at the right time and in a manner that reflects empathy and compassion. Many patients experience fear, distress and anxiety when receiving ‘bad news’, and these emotional reactions can be shaped by how healthcare providers present the information (Burton et al. 2021). Although positive communication is important, it should not involve misleading patients, as that can undermine trust between healthcare providers and patients. Instead, healthcare providers should prepare patients for difficult news and support them in managing their emotions afterward (Burton et al. 2021; Villagran et al. 2010). Therefore, we propose that mHealth applications should incorporate adaptive features that deliver information at appropriate times based on patient needs. For example, information could be provided through notifications or educational content when patients actively request it. Furthermore, the language used in educational materials should reflect empathy and compassion, ensuring that even sensitive or difficult information is communicated in a way that minimises distress. This can be achieved by integrating emotional intelligence into text responses and voice interactions, conveying warmth and understanding. For instance, the application could provide comforting prompts following the delivery of difficult news, assisting patients in processing the information gradually. In cases where patients experience significant distress, the application should offer real‐time access to healthcare professionals or mental health experts, such as via video calls or text‐based consultations, ensuring that patients can receive immediate human support following the receipt of challenging information.

### 4.3 Emotional and Social Needs

Among patients with prostate cancer, personal independence is often highly valued, and this intense pursuit of independent living can sometimes exceed other needs, even negatively impacting their health (Duner and Nordström 2005; Plath 2008). This tendency may be linked to the advanced age of prostate patients with prostate cancer (Yuen et al. 2007). Research from Pennsylvania State University shows that 77% of adult children believe their elderly parents are reluctant to accept advice or help with daily tasks (Heid et al. 2016), a refusal that may stem from their fear of losing independence. For many elderly individuals, technology helps in managing chronic diseases and maintaining their ability to live independently. Thus, mHealth could potentially become a crucial communication link between elderly patients and healthcare professionals. However, when designing mobile healthcare services, it is essential to respect the elderly's emphasis on ‘independence’. Specifically, when developing features related to patient interaction and information sharing, more personalised services may be necessary to support autonomy while enabling effective communication with healthcare professionals.

Emotional needs also play an important role in shaping the perceived barriers to mHealth adoption. Especially among elderly male patients, a strong sense of independence and reluctance to seek help led to feelings of confusion and helplessness when facing technical difficulties. Long‐term unresolved technical issues may exacerbate their emotional distress, further intensifying their resistance to mobile health solutions. This emotional distress not only deepens patients' anxiety but may also decrease their willingness to use mHealth. Therefore, in the design of mHealth tools, improving the availability and practicality of emotional support can better meet patients' emotional needs, reduce their resistance and promote behaviour change and wider adoption of mHealth tools.

Mindfulness and relaxation therapies serve as representative examples among a range of emotional support methods, playing a pivotal role in alleviating psychological pain and maintaining emotional health (Plath 2008). They help in reducing stress and trauma related to physical conditions such as pain, cancer, rheumatoid arthritis and other chronic diseases. This is particularly true for older adults, for whom mindfulness training for emotional regulation is highly suitable. However, our research indicates that there is a relatively low demand among patients for these types of emotional support therapies. When mentioned, patients often display disdainful or superficial expressions, quickly showing negative and rejecting attitudes. Studies show that when implementing mindfulness interventions in the elderly, dropout rates are quite high, averaging at 23% and in some cases even reaching 64% (O'Connor et al. 2014; Plath 2008). This general lack of interest may be a primary reason for these high dropout rates.

In our study, the initial analysis of this disinterest suggests that it is due to patients' lack of understanding about the benefits and significance of emotional support. They often view these therapies as deceptive and meaningless, leading to resistance. Providing clear and accessible educational materials is an effective way to address this issue. These materials should be integrated into the informational support offered via mHealth platforms, emphasising the proven benefits of emotional support therapies, particularly their role in alleviating stress and pain associated with prostate cancer. Additionally, offering patients greater flexibility and choice in utilising emotional support services can help to reduce resistance. By allowing patients to make informed decisions based on their preferences, they may feel more empowered and engaged, ultimately fostering greater acceptance of these therapies.

### 4.4 Self‐Management Needs

Multiple patients in our study mentioned that they often forget to take their medication and expressed a strong desire for mobile health applications to provide medication reminders. This observation aligns with the findings of previous qualitative research (McBride et al. 2020; Park et al. 2020). Earlier studies have also discovered that older individuals are more prone to medication non‐adherence compared with younger people (Chew et al. 2021). This is mainly due to cognitive decline, the complexity of their medication regimens and frequent changes in prescriptions. Implementing adaptive medication reminders, drug interaction warnings and personalised medication management plans on mobile devices have shown potential in enhancing self‐management of chronic diseases and improving medication adherence, thereby effectively increasing patients' compliance with their medication regimes (Al‐Arkee et al. 2021; Patel et al. 2013; Whitehead and Seaton 2016).

On another note, apart from treatment, patients undergoing ADT for prostate cancer often need to engage in personal exercises at home. A significant number of these patients, having undergone radical surgery, experience stress urinary incontinence (SUI) postoperatively (Braun et al. 2023). Pelvic floor muscle training (PFMT) has been proven to significantly improve the incontinence condition and quality of life in men after prostate cancer treatment (Jie et al. 2022). However, sustaining this training over a long period can be challenging for many patients. Therefore, they hope for reminders or guidance through mobile health applications (Jie et al. 2022). Therefore, to better support patients in self‐management, it is advisable to incorporate step‐by‐step video tutorials, progress tracking and motivational notifications into mobile health applications designed for patients with prostate cancer to support long‐term adherence to training regimens.

### 4.5 Integrating Needs, Facilitators and Barriers for mHealth Optimisation

The interplay between identified needs, facilitators and barriers provides a cohesive framework for guiding mHealth design and optimisation, particularly for patients with prostate cancer. Informational, emotional and self‐management needs serve as the foundational drivers for defining mHealth functionalities, addressing challenges such as providing accurate health information, psychological support, and tools for adherence to medication and rehabilitation schedules. Facilitators, such as family support and ease of use, play a crucial role in encouraging patients to initiate mHealth adoption. Additionally, fostering healthy competition among patients can enhance engagement and motivation, promoting sustained use of these applications. Conversely, barriers like technical complexity, which may lead to frustration and resistance, underscore the need for simplified operations and intuitive design to enhance usability. Together, these interconnected themes emphasise the importance of a user‐centred approach in mHealth development, ensuring that the tools are both effective in meeting patients' practical needs and adaptable for long‐term usability and acceptance. This integrated perspective not only addresses the immediate challenges faced by patients with prostate cancer but also informs strategies for improving adherence and optimising the overall user experience.

### 4.6 Strength and Limitations of the Work

This study focuses on prostate patients with cancer in China, a population for whom research on mHealth needs remains limited. Our findings provide several important insights.

First, we observed that Chinese patients with cancer, particularly those with prostate cancer, place a strong emphasis on TCM and wellness practices. This aspect has been rarely addressed in mHealth research conducted in Western contexts. Therefore, future studies on mHealth applications for Chinese patients should consider the influence of TCM‐related health beliefs on patient engagement and self‐management to enhance cultural adaptability and acceptance.

Second, patients with prostate cancer are predominantly elderly, yet their mHealth needs are often overlooked. It is commonly assumed that older adults struggle to adapt to mobile technology; however, our findings challenge this stereotype. The study reveals that older patients with prostate cancer demonstrate a higher‐than‐expected level of adaptability and acceptance towards mHealth tools. Some patients even exhibited notable adaptability, and with appropriate support, they were able to effectively use mHealth applications. These findings contribute to a better understanding of mHealth adoption among older adults and offer practical insights for designing age‐friendly mobile health solutions.

Furthermore, patients with prostate cancer are not only elderly but also predominantly male, a demographic whose mHealth needs have received little attention in existing research. This study provides new insights into the experiences, barriers and unmet needs of older male patients regarding mHealth use. In particular, it highlights their tendency towards emotional restraint and reluctance to seek assistance from their children, revealing a psychological paradox that influences their engagement with health management. These findings offer valuable perspectives on how to optimise mHealth solutions to better support the emotional well‐being of older male patients.

This study has several limitations. First, although 148 patients were invited, only 17 participated, leading to a low participation rate and potential selection bias. Participants may have been in better physical or mental health, or more adaptable, which could have influenced their views on mHealth. Efforts were made to ensure a diverse sample, but future studies should use broader recruitment methods (e.g., outreach across hospitals or patient networks) to ensure more representative results. Examining non‐respondents' health and tech adaptability could help understand this bias further. Second, this study was conducted in Shanghai, a more developed area, which may limit the applicability of the findings to other regions in China or internationally. Patients in Shanghai are likely to have better access to technology, which could influence their engagement with mHealth. In rural areas, patients may face different barriers, like limited smartphone access or lower digital literacy. Future studies should include a wider geographic sample. Lastly, this study lacks follow‐up interviews, so it cannot track changes in patients' needs over time. Longitudinal studies could offer insights into how patients' needs evolve during different treatment stages, such as the start of ADT versus long‐term maintenance.

### 4.7 Recommendations for Further Research

Future mHealth design should address not only patient needs—including information, emotional and social interaction, and self‐management—but also the scalability and adaptability of these interventions across diverse healthcare settings. Although this study focuses on patients with prostate cancer undergoing ADT in China, future research should explore its applicability to other chronic diseases requiring long‐term management.

Given regional disparities in digital literacy and healthcare accessibility, further studies should assess the feasibility of mHealth deployment in rural and under‐resourced areas. Cross‐country comparisons in similar developing healthcare systems could provide insights into cultural and systemic factors influencing adoption.

To ensure long‐term sustainability, longitudinal studies are needed to evaluate the sustained effects of mHealth on patient adherence, health outcomes and healthcare resource utilisation. Additionally, cost‐effectiveness analyses will be crucial for determining its viability for large‐scale implementation.

Finally, as healthcare professionals and family members play a key role in mHealth adoption, future research should explore their perspectives on integration into clinical workflows and patient support systems. Quantitative studies are also necessary to validate this study's findings in broader populations and assess the generalisability of mHealth‐driven interventions.

## 5 Implications for Policy and Practice

Our study highlights the critical role of interdisciplinary support, intuitive design and empathetic communication in addressing the barriers to mHealth adoption among elderly users. These findings underscore the need for targeted strategies to enhance user engagement and efficacy in mHealth applications. Building on these findings, this study also emphasises the necessity of providing tailored strategies to address the needs of key stakeholders, including healthcare providers, app developers and policymakers. For healthcare providers, actively engaging in interdisciplinary collaboration—particularly involving experts from both TCM and Western medicine—can ensure that patients receive balanced and evidence‐based information. Additionally, leveraging mHealth platforms to deliver empathetic, patient‐centred communication can alleviate anxiety and foster trust, especially when conveying complex or sensitive information. App developers, on the contrary, should prioritise the creation of intuitive and accessible interfaces that account for the cognitive and physical challenges faced by elderly users. By incorporating features such as personalised medication reminders, educational resources and clear navigation, developers can improve user satisfaction and promote sustained engagement. Finally, policymakers play a pivotal role in creating an environment conducive to mHealth adoption through initiatives that support digital literacy among the elderly, ensure equitable access to digital technologies and establish robust data privacy measures to build public trust. By integrating these strategies, healthcare systems can overcome the existing barriers to mHealth adoption and better serve the elderly population, enhancing both healthcare delivery and patient outcomes.

Looking ahead, we envision integrating mHealth applications with existing healthcare systems, particularly electronic health record (EHR) systems. By synchronising real‐time data from mHealth tools, such as symptoms, vital signs and other health metrics, healthcare providers can continuously monitor patient progress. This integration would enable timely adjustments to treatment plans and improve the accuracy of care. Furthermore, it could reduce the need for frequent hospital visits, alleviate healthcare resource burdens and enhance patient outcomes through remote monitoring and consultation. Ultimately, this integration would ensure more personalised, convenient and effective care, leading to higher patient satisfaction.

One significant barrier to mHealth adoption is the increased workload for healthcare professionals, particularly nurses and doctors. Given their already heavy responsibilities, adding tasks like patient training or responding to online queries could further strain their time. Elderly patients, in particular, may need additional support to use mHealth tools effectively, placing more pressure on healthcare providers. To mitigate this, mHealth applications should prioritise simplicity and ease of use, reducing the learning curve for both patients and healthcare professionals. Incorporating AI technologies, such as generative AI, can handle routine patient queries, alleviating healthcare workers from these repetitive tasks. Additionally, offering online resources and occasional training sessions will help patients, especially the elderly, become more confident with mHealth tools without overburdening providers. These strategies will facilitate smoother adoption of mHealth applications, improving patient care while minimising healthcare providers’ workloads.

## 6 Conclusion

In this study, we employed qualitative interviews to explore the needs and views of patients with prostate cancer undergoing ADT regarding mobile healthcare. The findings reveal key insights: although most patients have a positive attitude towards smartphones, only about half are willing to use mobile healthcare services. We found that support from healthcare professionals and family, competitive design features and a user‐friendly interface are major factors that facilitate the use of mobile healthcare. However, barriers include better self‐management among patients and low gerontechnology self‐efficacy, especially among older adults.

Patients expressed clear needs in terms of information requirements, emotional and social needs, and self‐management demands for mobile healthcare. For information support, they emphasised the importance of timely access to information and expressed a desire for the integration of traditional Chinese medicine knowledge. Regarding emotional support, patients preferred to maintain their independence and were reluctant to trouble family and friends; they also showed little interest in relaxation or mindfulness strategies for emotional support. Moreover, most were not inclined towards online interactions with other patients for support. In terms of self‐management, they hoped that mobile healthcare would provide medication reminders and reminders for rehabilitation exercises.

Overall, these findings provide valuable insights for the development and optimisation of mobile healthcare applications targeted at patients with prostate cancer, particularly in terms of enhancing patient engagement and satisfaction. By deeply understanding the specific needs and preferences of patients, we can better design and implement mobile healthcare solutions tailored to this particular patient group.

## Author Contributions

H.Y.: conceptualisation, data curation, data analysis, investigation, methodology, project administration, original draft. C.P.: data analysis, investigation, methodology, project administration, original draft. J.G.: data curation, data analysis, investigation, methodology, original draft. Y.Y.: conceptualisation, data analysis, funding acquisition, methodology, project administration, resources, supervision, review and editing. H.Y., C.P. and J.G. contributed equally. All authors reviewed and edited the manuscript and approved the final version of the manuscript.

## Ethics Statement

The study was approved by the ethical review of the School of Public Health and the School of Nursing of Shanghai Jiao Tong University.

## Conflicts of Interest

The authors declare no conflicts of interest.

## Supporting information


Table S1.

Data S1.

## Data Availability

The data that support the findings of this study are available on request from the corresponding author. The data are not publicly available due to privacy or ethical restrictions.
