# Supplementary material for: Perspectives and Requirements of Patients with Prostate Cancer on Mobile Health Interventions During Androgen Deprivation Therapy: A Descriptive Qualitative Study
Source: J Clin Nurs. 2025 Mar 9;35(5):2400–15. doi: 10.1111/jocn.17718 (PMC13068176; doi:10.1111/jocn.17718)
Supplement: Supplementary file 1 — Table S1. [file JOCN-35-2400-s002.docx]

Table S1: Demographic Data of Prostate Cancer Patients Undergoing ADT

| Patient | Pre-retirement Occupation | Monthly Household Income (CNY) | Other Treatments Received | Endocrine Therapy Medication |
| --- | --- | --- | --- | --- |
| N1 | Freelancer | Above 10,000 | Chemotherapy Radical Prostatectomy | Leuprorelin Acetate Microspheres for Injection |
| N2 | Worker | 8,000–9,999 | Radical Prostatectomy | Leuprorelin Acetate Microspheres for Injection Abiraterone Acetate Tablets combined with Prednisone Acetate Tablets |
| N3 | Civil Servant | 8,000–9,999 | Chemotherapy Radical Prostatectomy | Triptorelin Acetate for Injection Bicalutamide Tablets |
| N4 | Worker | 5,000–7,999 | / | Leuprorelin Acetate Microspheres for Injection Abiraterone Acetate Tablets combined with Prednisone Acetate Tablets |
| N5 | Worker | Above 10,000 | Radical Prostatectomy | Abiraterone Acetate Tablets combined with Prednisone Acetate Tablets |
| N6 | Worker | 3,000–4,999 | Radical Prostatectomy | Goserelin Acetate Sustained-Release Implant |
| N7 | Worker | Above 10,000 | Cryoablation Therapy for Prostate Cancer | Enzalutamide Tablets |
| N8 | Cadre | 8,000–9,999 | Radical Prostatectomy | Triptorelin Acetate for Injection |
| N9 | Worker | 3,000–4,999 | Radical Prostatectomy | Goserelin Acetate Sustained-Release Implant |
| N10 | Driver | Below 3,000 | Radical Prostatectomy | Goserelin Acetate Sustained-Release Implant Triptorelin Acetate for Injection Abiraterone Acetate Tablets combined with Prednisone Acetate Tablets |
| N11 | Worker | 8,000–9,999 | / | Triptorelin Acetate for Injection Abiraterone Acetate Tablets combined with Prednisone Acetate Tablets |
| N12 | Secondary School Teacher | 8,000–9,999 | Radical Prostatectomy Radiotherapy | Leuprorelin Acetate Microspheres for Injection Abiraterone Acetate Tablets combined with Prednisone Acetate Tablets |
| N13 | Cadre | Above 10,000 | Radical Prostatectomy Radiotherapy | Enzalutamide Tablets |
| N14 | Clerk | 5,000–7,999 | Bilateral Orchiectomy | Abiraterone Acetate Tablets combined with Prednisone Acetate Tablets |
| N15 | Professional Technician | Below 3,000 | Radiotherapy | Abiraterone Acetate Tablets combined with Prednisone Acetate Tablets |
| N16 | Professional Technician | 5,000–7,999 | / | Abiraterone Acetate Tablets combined with Prednisone Acetate Tablets |
| N17 | Farmer | Below 3,000 | / | Abiraterone Acetate Tablets combined with Prednisone Acetate Tablets |
